# A Robust Fully-Integrated Digital-Output Inductive CMOS-MEMS Accelerometer with Improved Inductor Quality Factor

**DOI:** 10.3390/mi10110792

**Published:** 2019-11-18

**Authors:** Yi Chiu, Hsuan-Wu Liu, Hao-Chiao Hong

**Affiliations:** 1 Department of Electrical and Computer Engineering, National Chiao Tung University, Hsin Chu 300, Taiwan; hchong@cn.nctu.edu.tw; 2 Taiwan Semiconductor Manufacturing Company, Hsin Chu 300, Taiwan; h3508666@gmail.com

**Keywords:** CMOS-MEMS, accelerometer, inductive, inductor, sprinductor, LC tank, oscillator, quality factor, digital output

## Abstract

This paper presents the design, fabrication, and characterization of an inductive complementary metal oxide semiconductor micro-electromechanical systems (CMOS-MEMS) accelerometer with on-chip digital output based on LC oscillators. While most MEMS accelerometers employ capacitive detection schemes, the proposed inductive detection scheme is less susceptible to the stress-induced structural curling and deformation that are commonly seen in CMOS-MEMS devices. Oscillator-based frequency readout does not need analog to digital conversion and thus can simplify the overall system design. In this paper, a high-Q CMOS inductor was connected in series with the low-Q MEMS sensing inductor to improve its quality factor. Measurement results showed the proposed device had an offset frequency of 85.5 MHz, sensitivity of 41.6 kHz/g, noise floor of 8.2 mg/√Hz, bias instability of 0.94 kHz (11 ppm) at an average time of 2.16 s, and nonlinearity of 1.5% full-scale.

## 1. Introduction

Micro-electromechanical systems (MEMS) accelerometers have been widely used in consumer electronic devices and automobiles. Typical MEMS accelerometers can be classified as capacitive [1,2,3,4], piezoresistive [5,6], convective [7], and inductive [8,9,10] types. Among these different types, the capacitive accelerometers have been attracting the most focus in both industry and academia due to their high sensitivity, insensitivity to temperature, and compatibility to the MEMS and complementary metal oxide semiconductor (CMOS) fabrication processes. Due to their miniature dimensions and, thus, their small signal levels, it is desirable to place the readout circuits close to the sensors to reduce the adverse effects of parasitics and noise. Monolithic integration of sensors and readout circuits on the same chip by using commercial CMOS processes is one of the best techniques to integrate CMOS circuits and MEMS sensors. In a monolithically integrated CMOS-MEMS capacitive accelerometer, the sensing capacitor is composed of a movable and a fixed electrode. Both electrodes are typically realized by stacking multiple metal and oxide layers of the backend CMOS processes [1,2,3,4]. Such structures are prone to deformation and curling due to the residual stress in the deposited films. The deformation of electrodes will change either the overlapping area or the gap between the electrodes and introduce uncertainty in its capacitance value and sensitivity. To overcome this problem, curl compensation frames [1] or pure oxide structures [2] were proposed. However, the deformation and curling of the sensor structures still could not be fully eliminated [2].

To alleviate the effect of residual stress on the sensor structure and its performance in the presence of structural deformation, this paper presents an inductive CMOS-MEMS accelerometer employing on-chip planar variable inductors as sensing elements. The main difference between inductors and capacitors as sensing elements is that capacitors have two physically distinct electrodes whereas inductors have only one coil loop. Therefore, stress-induced deformation changes only the curvature of the planar inductor coils, while the inductor areas and wire spacing remain largely constant. Therefore, the inductance of the planar coils and thus the characteristics of the sensor are more robust and consistent even in the presence of deformation.

CMOS-based inductive sensing has been applied to proximity sensing [11,12,13], magnetic biosensing [14], and tactile sensing [15]. The sensing inductors in these studies were either on-chip CMOS inductors [12,13,14], on-chip electroplated inductors [11], or off-chip printed circuit board (PCB) inductors [12,13]. The readout circuits were custom-designed on-chip CMOS circuitry [11,12,13,15] or a commercially available off-chip inductance conversion integrated circuit (IC) [14]. All of the above sensors are some forms of proximity sensors, i.e., they detect the distance between the sensing coil and another conducting or ferromagnetic object while the sensing coil remains fixed and undeformed. The requirement of two objects (coil and another conductor) or additional ferromagnetic materials in the sensors reduces the compatibility for monolithic CMOS integration.

In Liao’s research [8], bond wires and their parasitic inductance were used with on-chip circuits for acceleration detection. Although bond wires are readily available in standard packaged ICs, and no special materials are needed, the wire bonding process is much less precise than the CMOS process. Therefore, the variation of the sensor characteristics can be quite large. Inductive CMOS-MEMS accelerometers with integrated on-chip deformable inductors were first demonstrated in our previous works [9,10]. These are true monolithically integrated devices with no additional non-CMOS materials. The deformable sensing inductors are fabricated using standard CMOS processes with much higher precision compared to the bond wire inductors. Oscillator-based frequency readout was employed so that analog to digital conversion was not needed, and, thus, the overall system design could be simplified. However, it was found that the sensor performance could be limited by the low quality factors (Q factors) of the single-turn MEMS inductors in the LC-oscillator based readout design. This paper presents a new circuit design where a high-Q CMOS inductor is connected in series with the MEMS sensing inductor to improve the overall quality factor and, thus, the sensor performance. The proposed device was designed and fabricated by using a commercial 0.18 μm CMOS process plus post-CMOS release processing. The operation principle of the inductive accelerometer, the analysis of the effects of residual stress on the MEMS structures, the MEMS structure and CMOS circuit design, the CMOS-MEMS fabrication, and the sensor characterization are discussed in the following sections.

## 2. Operation Principle

**Figure 1:**
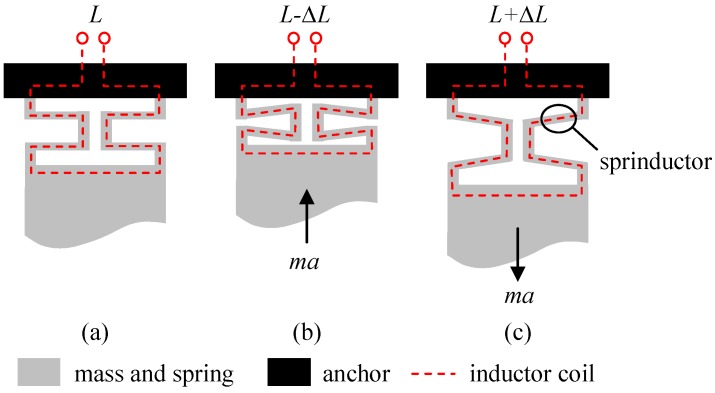
Schematic of proposed complementary metal oxide semiconductor micro-electromechanical systems (CMOS-MEMS) inductive accelerometer showing suspended mass and sprinductor: (**a**) Without acceleration, (**b**) compressed sprinductor and reduced inductance under acceleration, and (**c**) stretched sprinductor and increased inductance under acceleration.

The schematic of the inductive CMOS-MEMS accelerometer is shown in Figure 1 [10]. A proof mass, *m*, is suspended by four springs, *k*, and anchored to the substrate (Figure 1a). Embedded in the springs are metal routings forming closed electrical loops and effective inductors, *L*. Therefore, the springs and inductors share the same physical structures, and they are thus termed ‘sprinductors.’ Upon the action of an external acceleration, *a*, the inertial force, *ma*, deforms the sprinductors. In Figure 1b, the sprinductor is compressed and the surface area enclosed by the planar inductor coil is reduced. Since the inductance of a coil is related to the total enclosed magnetic flux when a current passes through it, the planar sprinductor with reduced area thus has a reduced inductance. Similarly, when the sprinductor is stretched by the inertial force, as shown in Figure 1c, its area and inductance are increased. If the deformation is small, the inductance change, ∆*L*, is approximately proportional to the external acceleration, *a*. Such sprinductors can be arranged on the two sides of the proof mass so that they undergo differential change of inductance, ±ΔL, when acted upon by the external acceleration. Such differential inductors can be used in differential readout circuits to enhance signals and suppress common-mode noise.

**Figure 2:**
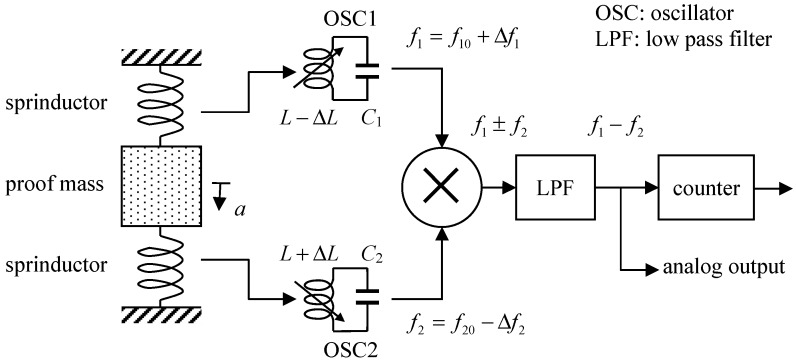
Readout circuit of proposed inductive accelerometer with digital output.

The principle of the readout circuit is shown in Figure 2. The inductance values of the sensing sprinductors are converted to frequency outputs by two LC tank oscillators with frequencies:

(1) $$f=f_{0}\mp \Delta f=1/2\pi \sqrt{(L\pm \Delta L)C}$$

where $f_{0}=1/2\pi \sqrt{LC}$ is the nominal oscillation frequency without acceleration and $\Delta f=(\Delta L/2L)f_{0}$ is the frequency shift due to external acceleration. As shown in Figure 2, the two oscillators are designed such that the capacitances, $C_{1}$ and $C_{2}$, in the LC tanks and, thus, the two oscillation frequencies, $f_{10}$ and $f_{10}$_,_ are different. The outputs of the two oscillators are mixed and low-pass filtered. Therefore, the frequency at the mixer output is:

(2) $$f_{1}-f_{2}=(f_{10}-f_{20})+(\Delta f_{1}+\Delta f_{2})\approx (f_{10}-f_{20})+2\Delta f$$

where $f_{10}-f_{20}$ is the frequency offset at the output for distinguishing between positive and negative input acceleration and 2Δf is the frequency output signal. An on-chip counter further converts the frequency output, 2Δf, into digital codes.

For adequate mechanical spring constants, *k*, the meandering sprinductors are realized by narrow and long metal/oxide composite beams that form single-turn inductors, *L*, as shown in Figure 1. Such MEMS inductors have low quality factors due to low inductance and high series resistance, $R_{s}$, of the loops. For example, the length and width of the segment beams in the sprinductor in this study are 395 μm and 5 μm, respectively. The inductance and resistance of such inductors obtained from the Coventorware (Coventor Inc., Raleigh, NC, USA) finite-element-method (FEM) simulation are listed in Table 1. The Q factor of the MEMS sprinductor at the 1.2 GHz oscillation frequency is only:

(3) $$Q_{L}=\omega L_{L}/R_{sL}=0.88$$

Such a low Q factor makes the oscillator design difficult and degrades its frequency stability and sensing resolution. Although it is possible to lower the series resistance and improve the Q factor by increasing the line width, *W*, of the metal routing, such a design simultaneously increases the width of the sprinductors and their spring constants, *k*. Furthermore, the series resistance is proportional to 1/*W* whereas the spring constant is proportional to *W*^3^. Since the sensitivity of a spring-mass accelerometer is inversely proportional to *k*, the improvement of Q by widening the sprinductor width is quickly diminished by the reduced sensitivity. In the current study, the width of the sprinductor is 5 μm according to the trade-off between inductance, spring constants, and fabrication yield. Instead of further increasing the sprinductor width, we propose to connect a high-Q CMOS inductor in series with the low-Q MEMS sprinductor to improve the total quality factor, $Q_{t}$. The high-Q inductor is implemented by a multi-turn coil in the top metal (M6) layer provided by the process design kits (PDK) from the CMOS manufacturer. The inductance, resistance, and quality factor of the low-Q MEMS sprinductor and the high-Q CMOS inductor used in this research are listed in Table 1. It can be seen that the total Q factor when the two inductors are connected in series is:

(4) $$Q_{t}=\omega (L_{L}+L_{H})/(R_{sL}+R_{sH})=4.26$$

at 1.2 GHz, showing a five-fold enhancement compared with the original low-Q sprinductor. The enhanced Q factor is expected to improve the oscillation stability and sensing resolution of the accelerometer.

## 3. Design and Simulation

The effect of residual stress on MEMS sensing structures such as comb fingers and sprinductors were investigated to manifest the advantage of inductive sensing over conventional capacitive sensing. The detailed design of the proposed accelerometer, including the MEMS sensing structure and the readout circuit, are discussed in the following sections.

### 3.1. Effect of Residural Stress on MEMS Sensing Structures

**Figure 3:**
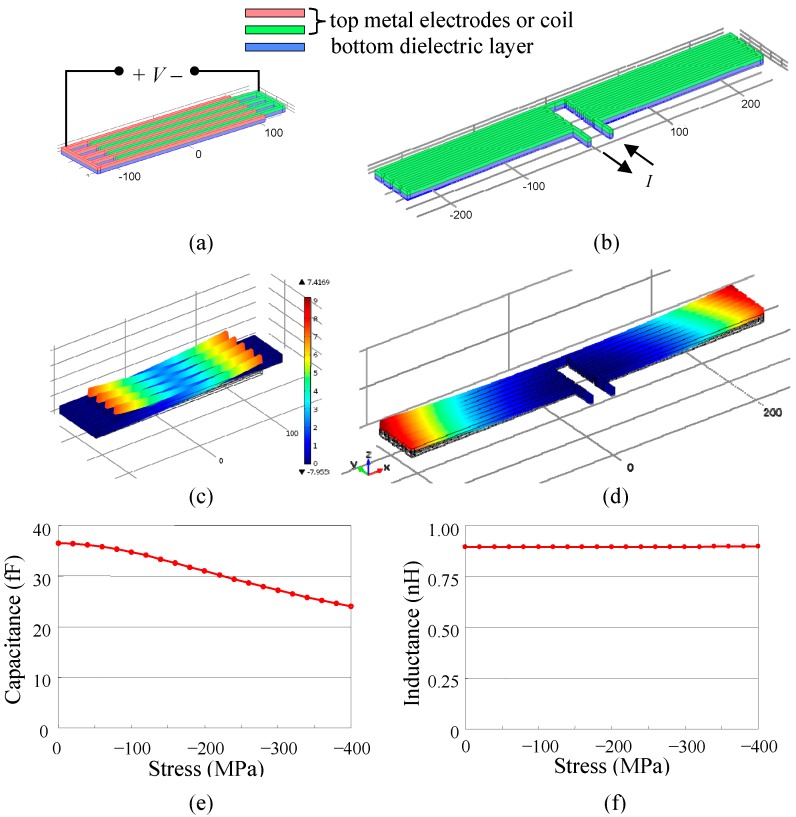
Finite-element-method (FEM) simulation of capacitance and inductance when subjected to residual stress induced deformation: (**a**) Comb finger capacitor, (**b**) meandering sprinductor, (**c**) deformed capacitor at −200 MPa residual stress, (**d**) deformed sprinductor at −200 MPa stress, (**e**) capacitance of comb finger capacitance vs. stress, (**f**) inductance of meandering sprinductor vs. stress. Note: (a)–(d) are shown to scale, (c) and (d) are shown with same color scale of total displacement.

Finite-element analysis by using Comsol 4.3b (COMSOL Inc, Stockholm, Sweden) was conducted to investigate the effect of structural deformation due to residual stress on the MEMS comb finger capacitance and meandering sprinductor inductance. To emulate the effect of residual stress in CMOS-MEMS, the comb fingers and sprinductors were modeled as bimorph structures, as shown in Figure 3a,b. The top layer was a conductor (aluminum) and the bottom layer was a dielectric (SiO_2_). Both layers were 5 μm thick. The length, width, and gaps of the comb fingers or coil segments were 230 μm, 4 μm and 4 μm, respectively. A residual stress was applied to the bottom layer to induce the deformation, as shown in Figure 3c,d. The capacitance and inductance of the deformed sensing structures were calculated using the FEM tool (Figure 3e,f). As shown in Figure 3c, the curling of the comb fingers significantly reduces the finger overlap area. Therefore, the capacitance is reduced by 34% at −400 MPa residual stress (Figure 3e). On the contrary, the sprinductor has a similar level of deformation (Figure 3d) but its inductance is only reduced by 0.2% at −400 MPa (Figure 3f). This confirms that, as a sensing mechanism, the inductance is much less sensitive and more robust when subjected to stress induced deformation as compared to the capacitance.

### 3.2. Accelerometer Structure Design

**Figure 4:**
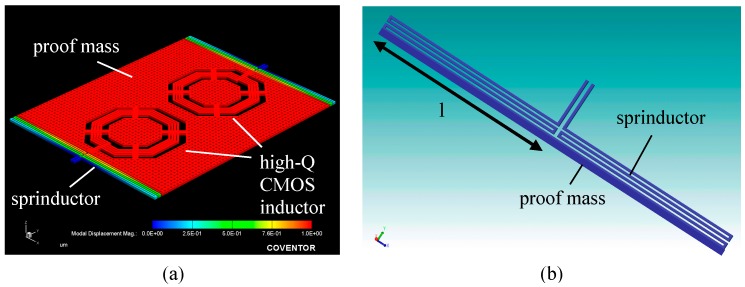
Solid model of MEMS structure: (**a**) whole device, (**b**) sprinductor.

The solid model of the MEMS sensing structure is shown in Figure 4a. The proof mass and sprinductors in the MEMS structure are composed of polySi-M1-M6 polysilicon-metal-oxide composite stacks with embedded tungsten via with a total thickness 10.14 μm. The area of the central proof mass is 800 × 900 μm. The high-Q CMOS inductor is embedded in the proof mass to save the chip area. Such a design also improves the Q factor of the CMOS inductor when the underneath silicon substrate is removed in the release process. The mass is suspended by 4 sprinductors at the corners. Mechanically, each sprinductor is a folded spring composed of 3 segment beams, as shown in Figure 4b, with a length (l) of 395 μm and a width of 5 μm. The total spring constant of the sprinductors was calculated by using the fixed-guided beam equation and was found to be 1.92 μN/μm.

**Figure 5:**
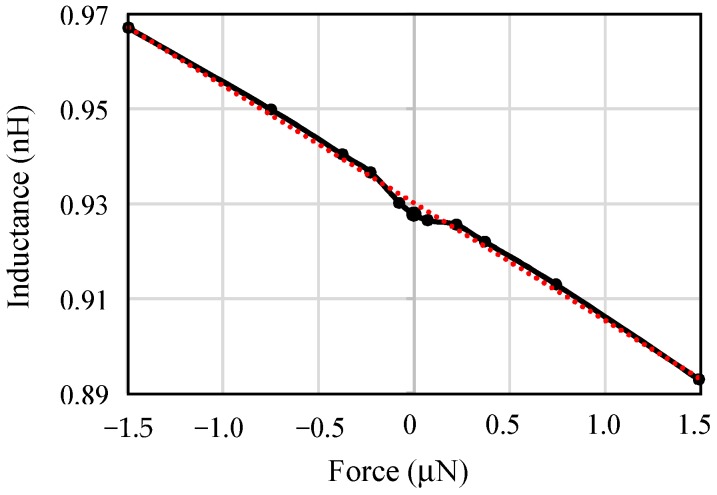
FEM simulation of inductance, *L*, under external force and deformation.

The inductance of the sprinductor under deformation due to external force was obtained from FEM simulation using Coventorware. Figure 5 shows that the sensing inductance of the sprinductor has a sensitivity of 2.5 × 10^−2^ nH/μN with respect to force. The sensitivity with respect to acceleration was found by taking into account the mass, *m*, to be 0.003 nH/g, or equivalently 3.2 × 10^−3^ (∆*L*/*L*)/g.

### 3.3. Oscillator Design

**Figure 6:**
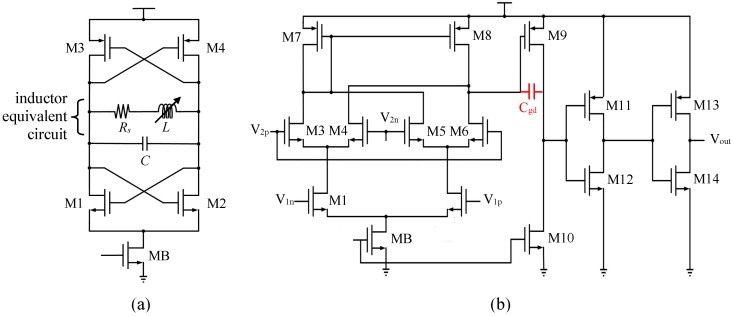
(**a**) LC tank oscillator, (**b**) Gilbert cell and single-end output buffer.

Figure 6a shows the LC oscillator that converts the sensing inductance value into frequency variation. The parallel LC tank that contains a series coil resistance, $R_{s}$, in the inductor equivalent circuit has a resonance frequency of:

(5) $$f_{0}=\frac{1}{2\pi \sqrt{LC}}\sqrt{1-\frac{R_{s}^{2}C}{L}}=\frac{1}{2\pi \sqrt{LC}}\sqrt{1-\frac{1}{Q^{2}}}$$

A MIM capacitor *C* = 1.67 pF was chosen so that the oscillation frequency was about 1.2 GHz. The positive-feedback transistors, M1~M4, provide effective negative resistance in parallel with the LC tank to compensate for the energy loss due to the series coil resistance, *R_s_*. The bias currents and aspect ratios of M1~M4 were designed according to the total resistive impedance at resonance found from post-layout extraction.

The outputs of the oscillators were connected to common-sources buffers before the mixing. Since the two inputs of the mixer need to be biased at different levels, the output common mode of the buffers for the two differential oscillators were biased at 0.9 V and 1.4 V, accordingly. The different output bias of the buffers intrinsically results in different load capacitance for the two oscillators. Therefore, the two oscillators will automatically oscillate at slightly different frequencies, $f_{10}$ and $f_{20}$, as required by Equation (2). It is noted that additional buffers were implemented and connected in parallel to the main signal path so that the analog oscillation signals could be observed directly by external instruments, as shown in Figure 2.

### 3.4. Mixer, Low-Pass Filter, and Counter Design

Figure 6b shows the Gilbert cell mixer in this study. The differential outputs of the two oscillators are connected to M1/M2 and M3~M5, respectively. The differential output current of the mixer is converted by M7 and M8 to a single-ended signal. It is further amplified to a rail-to-rail signal by the common-source amplifier, M9 and M10. The gate-drain capacitance, C_gd_, of M9 is amplified by the Miller’s effect and presents a large capacitive load at the mixer output that limits the effective bandwidth and removes the sum-frequency component in the mixer. Thus, only the difference frequency in Equation (2) passes through the output buffer. The buffer, M11~M14, further amplifies the signal to drive the external instruments or next-stage circuits. The output of the buffer is fed to an 18-bit on-chip synchronous counter for a typical mixer output frequency of 100 MHz and sampling frequency of 500 Hz.

## 4. Results and Discussion

The proposed accelerometer was fabricated using a standard CMOS process followed by post-CMOS release processing. After the MEMS structure was released, its resonance frequency was measured to ensure successful releasing. The oscillators were tested first when the device was at rest. The accelerometer was then tested by using a rotation table and a shaker for static and dynamic characterization, respectively.

### 4.1. CMOS and Post-CMOS Fabrication

**Figure 7:**
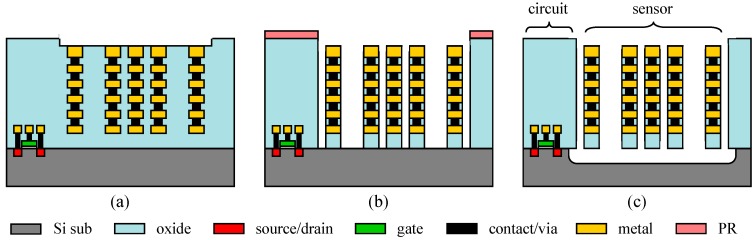
Fabrication process of proposed CMOS-MEMS accelerometer.

**Figure 8:**
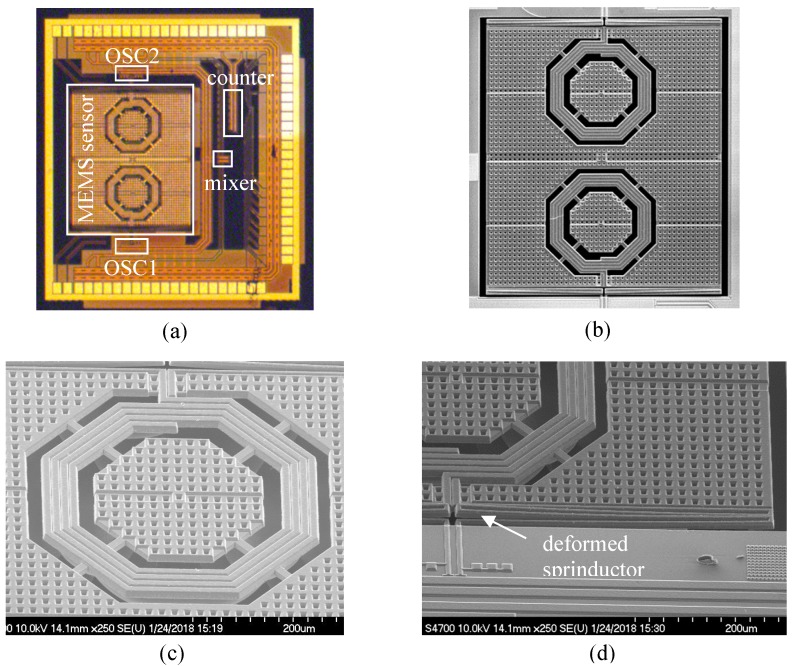
(**a**) Chip micrograph before release, (**b**) scanning electron micrograph of MEMS structure after release, (**c**) high-Q CMOS inductor embedded in proof mass, (**d**) deformed sprinductor.

**Figure 9:**
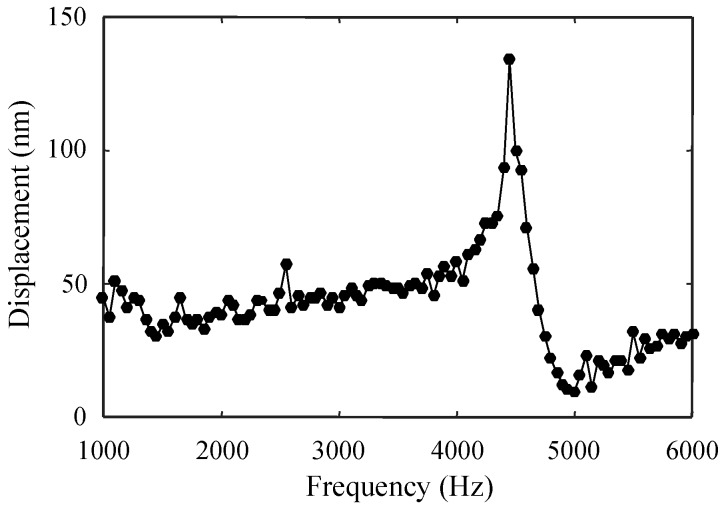
Resonance frequency measurement of release MEMS structure.

The device was first fabricated by a 0.18 μm 1P6M commercial CMOS process followed by post-CMOS dry-etching releasing provided by the Taiwan Semiconductor Research Institute (TSRI), Taiwan, R.O.C. [16]. Figure 7a depicts the cross section of a CMOS device as received from the CMOS foundry. In the post-CMOS release process, a photoresist (PR) layer was first applied to cover the CMOS circuit areas so that they would not be affected by the following etching processes (Figure 7b). In Figure 7b, CF_4_-based anisotropic reactive ion etching (RIE) was applied to remove the sacrificial oxide around the sensor structures. Subsequently, SF_6_-based isotropic RIE was used to remove the silicon substrate and release the sensor structures (Figure 7c). Figure 7c also shows the released sensor structures, such as the proof mass and the sprinductors, which are typically composed of the materials in the backend CMOS process, including oxide, aluminum metal, and tungsten via. Figure 8 shows the micrographs of a fabricated and released device. Figure 8c shows that the sprinductors are deformed due to the residual stress. The tip deformation was measured by a WKYO NT-1100 (Bruker Inc, Tucson, AZ, USA) white light interferometer, and was found to have an average value of 10.3 μm, corresponding to a radius of curvature of 8 mm. Such a large deformation would seriously affect the capacitance values and the sensor characteristics if comb finger sensing capacitors were employed. The in-plane resonance frequency of the released MEMS structure was measured by a PSM-1000 planar motion analyzer (Polytec GmbH, Waldbronn, Germany)). Figure 9 shows that the resonance frequency is 4.45 kHz with a mechanical Q factor of about 37.

### 4.2. Accelerometer Characterization

**Figure 10:**
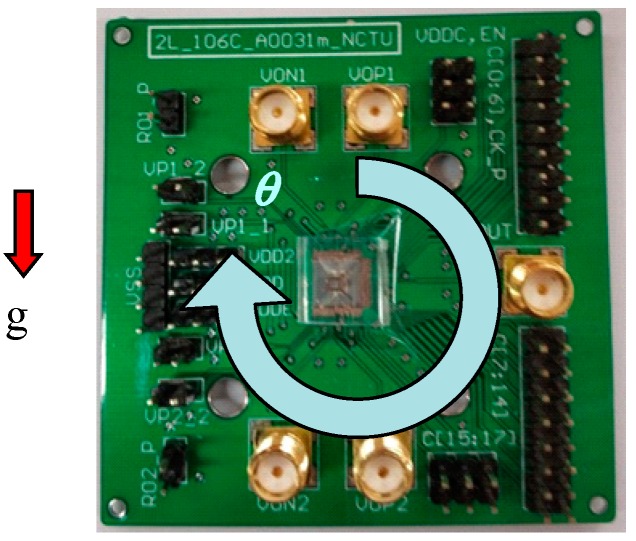
CMOS-MEMS accelerometer die mounted on a printed circuit board (PCB) for electrical testing.

The released CMOS-MEMS accelerometer was mounted in the center of a printed circuit board (PCB), as shown in Figure 10, and tested for both circuit functions and acceleration sensitivity.

#### 4.2.1. Oscillator Test

**Figure 11:**
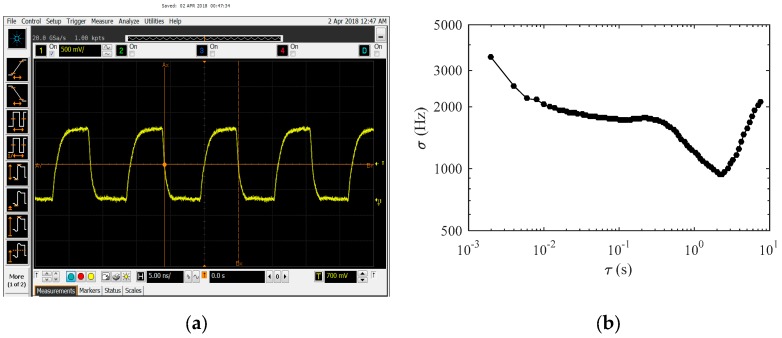
(**a**) Mixer output waveform, (**b**) Allan’s deviation at counter output.

The oscillation frequencies of the two oscillators were measured by a Keysight N9030B signal source analyzer (Keysight Inc, Santa Rosa, CA, USA) and were found to be 1.484 GHz and 1.382 GHz, respectively, with a frequency difference of 102 MHz. The measured frequency at the analog output of the mixer was 92.3 MHz, as shown in Figure 11a. The measured frequencies agree well with the design value. The slight discrepancy between the oscillator frequency difference and the mixer frequency is attributed to the different buffers that drive the mixer and the external instruments.

The frequency output of the on-chip counter was capture by a Keysight 16902B logic analyzer (LA) (Keysight Inc., Santa Rosa, CA, USA) at a sampling rate of $f_{s}$ = 500 Hz for 16 s. The frequency stability of the counter output was analyzed for Allan’s deviation. As shown in Figure 11b, the frequency bias instability is 0.94 kHz (11 ppm) at average time *τ* = 2.16 s.

#### 4.2.2. Static Acceleration Test

**Figure 12:**
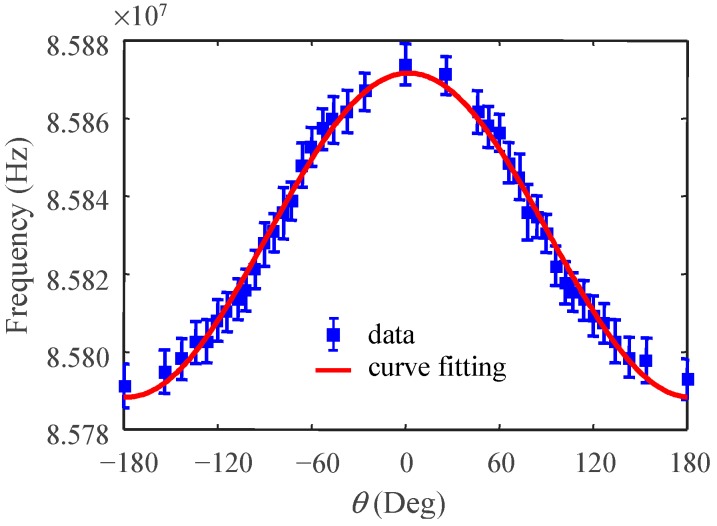
Static acceleration test.

The PCB in Figure 10 was mounted on a rotation stage to measure the component of the gravitational acceleration projected onto the sensing axis, *g*cos𝜃. Figure 12 shows the measured counter frequency, *f*(𝜃), vs. rotation angle, 𝜃, at a sampling rate of $f_{s}$ = 500 Hz. Curve fitting shows:

(6) $$f(\theta )=4.16\times 10^{4}\cos(\theta )+8.58\times 10^{7},R^{2}=\;0.9889$$

Therefore, the offset frequency, $f_{0}$, and sensitivity, $S_{f}$, of the accelerometer are 85.8 MHz and 41.6 kHz/g, respectively. The average standard deviation of frequency in Figure 12 is $\sigma_{f}$ = 5.4 kHz. Therefore, the noise floor of the accelerometer is:

(7) $$\mathrm{noise}\;\mathrm{floor}\;=\frac{\sigma_{f}/S_{f}}{\sqrt{f_{s}/2}}=8.2\;\mathrm{mg}/\sqrt{\mathrm{Hz}}$$

#### 4.2.3. Dynamic Acceleration Test

**Figure 13:**
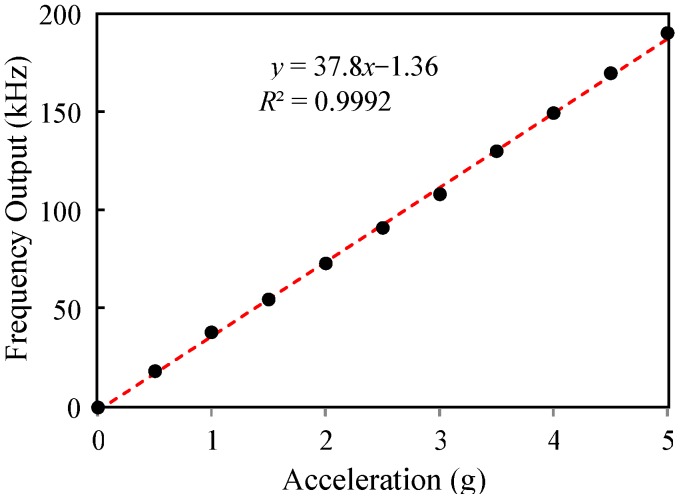
Dynamic acceleration test.

The dynamic acceleration test was performed by mounting the PCB in Figure 10 on an electrodynamic shaker (2007E, The Modal Shop, Inc., Cincinnati, OH, USA). A reference accelerometer (PCB 352C65, PCB Piezotronics, Inc., Depew, New York, NY, USA) was mounted coaxially to calibrate the vibration levels. The excitation vibration frequency was 50 Hz, and the sensor output was recorded at $f_{s}$ = 500 Hz. Figure 13 shows the signal amplitude vs. vibration levels. The sensitivity is 37.8 kH/g, and the nonlinearity is 1.5% full-scale (FS). The discrepancy between the static and dynamic sensitivities is attributed partially to the different test setup.

The performance comparison of the proposed device and other CMOS or CMOS-MEMS accelerometers in the literature is shown in Table 2. It can be seen that the current device has much improved performance as compared with our prior work without the series high-Q inductor [9]. The other performance parameters are also comparable to similar devices in the literature.

## 5. Conclusions

This paper presents the design, fabrication, and characterization of an inductive CMOS-MEMS accelerometer with on-chip digital output based on LC oscillators. FEM analysis confirms that the inductive detection scheme is much more robust in the presence of residual stress induced deformation as compared with capacitive detection. A high-Q CMOS inductor was connected in series with the low-Q MEMS sprinductor to improve its quality factor. The measurement showed that the proposed device had an output offset frequency of 85.5 MHz, sensitivity of 41.6 kHz/g, noise floor of 8.2 mg/√Hz, bias instability of 0.94 kHz (11 ppm) at average time 2.16 s, and nonlinearity of 1.5% full-scale. The performance compares well with similar CMOS or CMOS-MEMS accelerometers in the literature.

## Figures and Tables

**Table 1:** Inductor parameters at 1.2 GHz.

Item	*L* (nH)	$R_{s}\;(\Omega )$	*Q*
Low-Q MEMS sprinductor	$L_{L}$ = 0.92	$R_{sL}$ = 7.92	$Q_{L}$ = 0.88
High-Q CMOS inductor	$L_{H}$ = 8.91	$R_{sH}$ = 9.34	$Q_{H}$ = 7.19
Total inductor	$L_{t}$ = 9.83	$R_{st}$ = 17.26	$Q_{t}$ = 4.29

**Table 2:** Performance comparison of monolithic CMOS accelerometers.

CMOS Tech. (𝛍m)	Type ^1^	Sensing Element	$f_{0mech}{\;}^{2}\;(\mathrm{kHZ})$	$f_{0elec}{\;}^{3}\;(\mathrm{GHz})$	Sensitivity (/g)	Noise Floor (mg/√Hz)	Nonlinearity (%FS)	Output ^4^	Reference
0.35	C	on-chip	8.8	NA ^5^	105 mV	0.4	1.0	A	[2]
0.18	C	on-chip	4.7	NA ^5^	191 mV	0.35	1.0	A	[3]
0.18	C	on-chip	7.0	1.9	3.6 MHz	0.2	1.2	A	[4]
0.13	I	bond wire	3.1	2.1	10 kHz	80	-	A	[8]
0.18	I	on-chip	6.6	2.0	22 kHz	460	11	A	[9]
0.18	I	on-chip	4.5	1.4	42 kHz	8	1.5	A/D	This work

^1^ C: capacitive, I: inductive; ^2^ mechanical resonance frequency; ^3^ electrical oscillation frequency; ^4^ A: analog, D: digital; ^5^ chopper-based readout.
